# Insecticidal potential of five medicinal plants: An *In Vitro* evaluation and molecular docking analysis of *Artemisia absinthium*

**DOI:** 10.1371/journal.pone.0325959

**Published:** 2025-07-01

**Authors:** Mohd Yaseen, Mudasir Gani, Shabir Ahmad Ganai, Baseerat ul Ann, Fehim Jeelani Wani, Mohd Ayoub Mantoo, Khalid Rasool, Rashid Mumtaz Khan

**Affiliations:** 1 Division of Entomology, Faculty of Agriculture, Sher-e-Kashmir University of Agricultural Sciences and Technology of Kashmir, Jammu & Kashmir, India; 2 Division of Basic Sciences & Humanities, Faculty of Agriculture, Sher-e-Kashmir University of Agricultural Sciences and Technology of Kashmir, Jammu & Kashmir, India; 3 Division of Agricultural Economics & Statistics, Faculty of Agriculture, Sher-e-Kashmir University of Agricultural Sciences and Technology of Kashmir, Jammu & Kashmir, India; 4 Division of Horticulture, Faculty of Agriculture, Sher-e-Kashmir University of Agricultural Sciences and Technology of Kashmir, Jammu & Kashmir, India; 5 Department of Chemistry, College of Science, Qassim University, Buraidah, Saudi Arabia; PMAS Arid Agriculture University: PMAS-Arid Agriculture University Rawalpindi, PAKISTAN

## Abstract

The Kashmir range of North-Western Himalayas harbours a large number of medicinal plants that have insecticidal, antifeedent or insect repellent properties. Herein, we evaluated the insecticidal activity of five medicinal plants including *Artemisia absinthium, Acorus calamus*, *Digitalis purpurea*, *Plectranthus rugosus* and *Achiella millefolium* against *Corcyra cephalonica, Sitophilus oryzae* and *Helicoverpa armigera*. The highest *in vitro* insecticidal activity was observed with the *A. absinthium* plant extract against the test insects. Following this, *A. absinthium* extract was assessed through high resolution liquid chromatography mass spectrometry (HR-LCMS). The small molecules thus identified from the *A. absinthium* extract were flexibly docked against the active site of acetylcholinesterase *(Helicoverpa armigera)* keeping the known inhibitor malaoxon as the reference. Four molecules encompassing kaempferol, diosmetin, 1,7-Bis(4-hydroxyphenyl) heptan-3-one and NP-021018 explored from the defined extract manifested the highest binding affinity than malaoxon. As kaempferol evinced the maximum binding activity towards acetylcholinesterase thus, the insecticidal activity of *A. absinthium* extract may be attributed to this molecule.

## Introduction

Medicinal plants have global distribution and are an important source of compounds with congenital insecticidal properties [1–3]. The utilization of medicinal plants as insecticides is increasing as they are affordable and easily accessible to the farming community, besides being target specific and eco-friendly [4]. The Himalayan region comprised of India, China, Nepal, Myanmar, Bhutan, Bangladesh, Afghanistan and Pakistan is the hotspot of important medicinal plant species. About 200 plants from the families Meliaceae, Rutaceae, Asteraceae and Canaliaceae have been studied extensively for their insecticidal properties and are being developed as green pesticides [5]. There are about 8000 species of flowering plants in India and about 2000 plant species are having medicinal properties [6]. The Northwest Himalayan region comprises two Union territories (Jammu and Kashmir & Ladakh) and two states of the Indian Republic namely Himachal Pradesh and Uttarakhand harbours a large number of medicinal plants [7]. Several medicinal plant species such as *Azadirachta indica, Artemisia absinthium*, *Allium sativum, Mentha pulegium*, *Nerium oleander*, *Citrullus colocynthis*, *Ocimum basilicum*, *Origanum majorana, Laurus nobilis*, *Myrtus communis* etc. have been reported to have insecticidal or insect repellent properties [8]. The neem is the model plant-based insecticide that is derived from the plant *Azadirachta indica* and is safe for the humans, environment and other non-target organisms [9]. There are around 400 species of *Artemisia* reported from temperate regions of the world and many show antifungal, antibacterial, antioxidant and cytotoxic activities [10,11]. In the Himalaya, 19 species of *Artemisia* have been reported to have medicinal properties [12]. The *Artemisia absinthium* is a medicinal plant of great importance and is naturally distributed in the Jammu and Kashmir at an altitude range of 1500–2700 meters above sea level [13].

The damage caused by insect pests is one of the major limiting factor in global food production and the insects are reported to consume about 5–20% of major grain crops [14]. The chemical insecticides are frequently employed to manage insect pests, but their toxicity puts the health of farmers, animals and food consumers at risk. Further, the insects have also developed resistance against many classes of insecticides. The botanical insecticides are becoming more popular due to their eco-friendly nature and safety to non-target organisms including beneficial insects such as biocontrol agents, pollinators etc. [15]. The plant extracts or their pure compounds control insect pests in several ways, including antifeedant effect, growth inhibitors, toxicity, alteration in reproductive behaviour and fertility [16]. The forests of Kashmir are valued for their rich medicinal plant species such as *Artemisia absinthium* (wormwood, Asteraceae), *Achillea millefolium* (common yarrow, Asteraceae), *Acorus calamus* (sweetflag, Acoraceae), *Digitalis purpurea* (foxglove, Plantaginaceae), *Plectranthus rugosus* (Sulai, Lamiaceae) etc that represent a significant pool for the identification of molecules with insecticidal properties and the development of botanical insecticides. Although the insecticidal properties of few medicinal plants from Kashmir region have been determined, the identification of bioactive compounds using molecular techniques and their scientific validation has not been conducted hitherto. High Resolution – Liquid Chromatography Mass Spectrometry (HR-LCMS) enables the identification of bioactive compounds in plant extracts by separating, detecting and characterizing compounds based on their mass to charge ratio. HR-LCMS integrates two techniques high performance liquid chromatography (HPLC) and mass spectrometry (MS) [17]. While HPLC is considered an analytical technique, the MS is regarded as the analysis cum detection technique. Molecular docking complements this by predicting the interactions of bioactive compounds with target proteins, thereby helping to validate their potential insecticidal effects at the molecular level. In this context, the insecticidal activity of five different extracts of temperate medicinal plants namely *Artemisia absinthium, Achillea millefolium*, *Acorus calamus, Digitalis purpurea* and *Plectranthus rugosus* was conducted against rice moth [(*Corcyra cephalonica*), (Lepidoptera: Pyralidae)], rice weevil [(*Sitophilus oryzae*), (Coleoptera: Curculionidae)] and gram pod borer [(*Helicoverpa armigera*), (Lepidoptera: Noctuidae)]. This was followed by the identification of small molecules from the most promising extract using HR-LCMS and their subsequent flexible docking against the acetylcholinesterase enzyme of *Helicoverpa armigera*.

## Materials and methods

### Medicinal plants

The *Achillea millefolium* (common yarrow), *Artemisia absinthium* (wormwood), *Acorus calamus* (sweetflag) and *Digitalis purpurea* (foxglove) plants were collected from the Regional Cum Facilitation Centre, FoA, Wadura. The sampling location, Faculty of Agriculture, SKUAST-K, Wadura, Sopore, is situated at approximately 34.298676° N latitude and 74.470146° E longitude. The *Plectranthus rugosus* plants were collected from Banihal, Jammu and Kashmir, located at approximately 33.4394° N latitude and 75.1944° E longitude, at an altitude of 2832 metres. The plants were collected during May, 2023 and brought to the laboratory for further processing.

### Insects

The culture of test insects namely Rice moth (*Corcyra cephalonica*), Rice weevil (*Sitophilus oryzae*), and Gram pod borer (*Helicoverpa armigera*) were established in the Laboratory of the Division of Entomology, FoA, Wadura as per the standard methods as detailed below:

### Establishment of Laboratory culture of *Corcyra cephalonica* (Lepidoptera: Pyralidae)

The *Corcyra* rearing wooden boxes were sterilized in hot air oven at 100^0^C for 1 hour. Then 2.5 kg sterilized crushed maize (100^0^C for 1 hour), 50 g of chickpea flour, 5 g of yeast, 1 g of wettable Sulphur and 0.05 g of Streptomycin sulphate was added in each box. All the above ingredients were properly mixed and 1 cubic centimetre (1 cc) *Corcyra* eggs were sprinkled on top of the box. The rearing boxes were tightly closed with the lid, labelled and kept in the laboratory at 28 ± 2^0^C temperature and 75 ± 5% Relative Humidity (R.H.). The *Cocryra* adults started emerging after 45–50 days and the adult moths were collected and transferred to the egg laying chamber. The adults were provided with 20% honey + Vitamin E solution as food using cotton swabs that were kept hanging inside the egg laying chamber. The eggs were collected, sprinkled in the rearing boxes and later the 2^nd^ instar larvae were collected for experiments.

### Establishment of laboratory culture of *Sitophilus oryzae* (Coleoptera: Curculionidae)

The culture of the rice weevil was initiated by collecting the adult weevils from the infested seed samples from the maize seed godown at Sopore, Kashmir. For mass multiplication, the live adults of rice weevil were transferred in plastic jar (2 kg capacity) containing sterilized maize and the jar was covered with muslin cloth with the help of rubber bands. Later the larvae were collected for experiments.

### Establishment of laboratory culture of *Helicoverpa armigera* (Lepidoptera: Noctuidae)

The laboratory culture of *H. armigera* was established as per the methods described by Manzoor et al. (2023) [18]. Briefly, the larvae were collected from tomato and chick pea plants at experimental farm FoA, SKUAST-K, Wadura. The 1^st^ instar larvae were reared on Rumex (*Rumex obtusifolius*) and the later instars were reared on the semi-synthetic diet and transferred into multi-well plates or individual glass vials to avoid cannibalism. The pupae were transferred into plastic jars for adult emergence and the adults were released into oviposition cages (45 cm length: 30 cm diameter) that were lined internally with muslin cloth for egg laying. The culture was maintained at 28 ± 2°C, 60% R.H., and 16L: 8D photoperiod.

### Preparation of extracts

The extracts were prepared from the entire plant as per the methods described by Nascimento *et al*. (2022) [19]. The plants were first cleaned with a tap water to remove dust and dirt and then the fresh weight of each individual plant was recorded. The plants were dried in a hot air oven at 60–70 °C for 2–3 hours, after which the dry weights were recorded. The percentage of water content lost during the drying process was 84.00%, 84.44%, 65.00%, 75.76% and 78.79% for *Achillea millefolium*, *Artemisia absinthium*, *Acorus calamus*, *Digitalis purpurea*, and *Plectranthus rugosus*, respectively. The dried plants were powdered in the grinder individually and the dry powder was packed in the glass bottles and kept in the laboratory for use in the experiments. An amount of 10 grams of dry powder from each plant species was mixed individually with 100 ml of distilled water using a simple blender. The suspensions were then subjected to maceration by placing them on a shaking incubator for 24 hours. After maceration, the supernatants (extracts) were collected and stored at 4 °C in the laboratory for further use. Later the extracts were used for the determination of insecticidal activity and identification of small molecules using HR-LCMS.

### Assessment of insecticidal activity

The insecticidal properties of individual extracts were determined against Rice moth (*Corcyra cephalonica*), Rice weevil (*Sitophilus oryzae*) and Gram pod borer (*Helicoverpa armigera*) though bioassays. The bioassays were carried out at three different concentrations (5%, 10%, 15%) with five extracts, two positive controls (Deltamethrin 2.5 SC 1 ml/L and Lambda-cyhalothrin 4.9 SC 1.6 ml/L) and one negative control (Distilled water) totalling 18 treatments in three replications. The 2^nd^ instar larvae of each insect species were collected from laboratory rearing and 10 larvae of *C. cephalonica, S. oryzae* and *H. armigera* were kept in Petri dish individually. Then three different concentrations (5%, 10%, 15%) of each medicinal plant were prepared and 10 µl was sprayed on each larvae using a syringe. The *C. cephalonica, S. oryzae* larvae were reared in Petri plate and proper feed was provided as described above. The *H. armigera* larvae were reared individually in glass vials to avoid cannibalism. Each treatment was replicated three times with 10 larvae in each replicate. The number of dead larvae was counted 24, 48 and 72 hours post application and the mortality (%) was calculated using the formulae.


$$\begin{array}{c} Mortality\;(\%)=\frac{Number\;of\;dead\;larvae}{Total\;number\;of\;larvae}\times 100 \end{array}$$

### HR-LCMS of *Artemisia absinthium* extract

To identify small molecules from the extract of *Artemisia absinthium*, a high-resolution Orbitrap liquid chromatography–mass spectrometry (HR-LCMS) system was employed. Chromatographic separation was performed using a Hypersil GOLD column (150 × 2.1 mm, 1.9 µm, Thermo Scientific) [20]. The elution was carried out in gradient mode, with solvent A consisting of 0.1% formic acid in Milli-Q water and solvent B being acetonitrile. A sample injection volume of 2–5 µL was used, consistent with standard parameters for Orbitrap-based workflows using narrow-bore columns. The analysis was performed using a Q-Exactive Plus Biopharma mass spectrometer (Thermo Scientific). Xcalibur software (version 4.2.28.14) was used for data acquisition, and Compound Discoverer 3.2 SP1 was employed for data processing and small molecule identification.

### 3D-Model Generation, refinement and validation

The amino acid sequence of Helicoverpa armigera acetylcholinesterase enzyme was taken from the UniProt (primary accession number: Q8MX85). From this sequence the three-dimensional structure was generated using a tool based on hierarchical approach known as Iterative Threading ASSEmbly Refinement (I-TASSER) [21]. Among the five models predicted through I-TASSER the correct one was selected for further refinement. The model was refined using atomic scale and high-resolution refinement algorithm commonly known as ModRefiner [22]. The model was validated through different methods. While the stereochemical quality of model was estimated through PROCHECK program, it’s 3D-1D compatibility was explored through Verify3D [23,24].

### Active site specification and molecular docking

The ligand-binding site of refined model was predicted through COACH [25]. The refined model was given as an input and the final site was explored through a standard procedure. The coordinates of various molecules confirmed from the *A. absinthium* such as Kaempferol, Pipecolic acid, Diosmetin, 1, 7-Bis (4-hydroxyphenyl) heptan-3-one, NP-021018 were retrieved from PubChem [26]. The PubChem CID of these molecules is 5280863, 849, 5281612, 14608480 and 125416500, respectively. We used Malaoxon, the oxidation product of Malathion (PubChem CID: 15415) as the positive control [27]. Molecular docking of these ligands was performed in flexible mode using the CB-Dock2 [28]. The refined model of acetylcholinesterase enzyme was used as receptor and the above mentioned (six) molecules were employed as ligands. This tool as a default predicts five sites and docks the ligands in them. The optimal pose of the ligand is recorded in each site. Based on the active site information of COACH, the best pose of the ligand in the correct active site was chosen and its corresponding docking score was recorded for further analysis.

### Statistical analysis of data

Mortality data were analyzed using one-way analysis of variance (ANOVA), followed by post hoc tests for multiple mean comparisons using SPSS (Version 19). Docking scores of the five *Artemisia absinthium* compounds and the positive control (Malaoxon) were compared based on their binding affinities at the predicted active site. Statistical significance was determined using one-way ANOVA with a *p*-value < 0.05 considered significant. HR-LCMS analysis was conducted using a Q-Exactive Plus Biopharma mass spectrometer (Thermo Scientific), with data acquisition through Xcalibur software (version 4.2.28.14) and data processing via Compound Discoverer 3.2 SP1.

## Results

### Concentration- response bioassays

**Fig 1:**
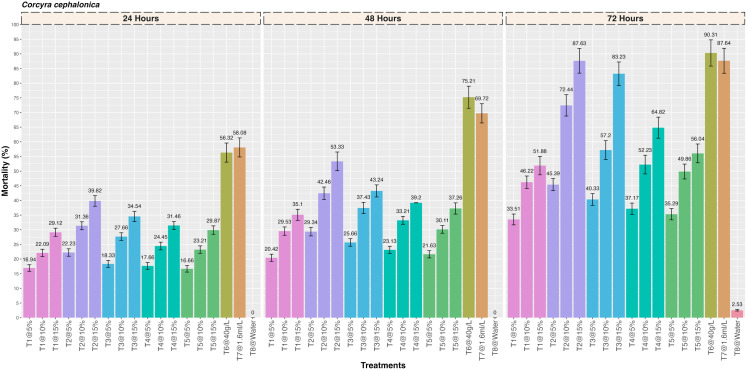
Insecticidal activity of five different medicinal plant extracts against rice moth, *Corcyra cephalonica* by contact toxicity at different concentrations.

The results revealed that the five different medicinal plant extracts caused mortality in the *C. cephalonica* larvae at three different concentrations (5%, 10% and 15%), the differences being significant between the treatments (Fig 1). It was found that there was a significant increase in the mortality with increase in the concentration of each plant extract and the mortality observed with the *Artemisia absinthium* plant extract, *Acorus calamus, Digitalis purpurea, Plectranthus rugosus* and *Achiella millefolium* was 39.82 ± 1.79, 34.54 ± 1.76, 31.46 ± 1.36, 29.87 ± 1.45 and 29.12 ± 1.41% at 24 hrs after treatment, respectively (F = 7.27; df = 17,36; p = < 0.001). Similarly, the mortality observed at 48 hrs after treatment was 53.33 ± 3.19, 43.24 ± 2.06, 39.20 ± 0.04, 37.26 ± 1.91 and 35.10 ± 1.89% for *Artemisia absinthium*, *Acorus calamus, Digitalis purpurea, Plectranthus rugosus* and *Achiella millefolium* plant extract, respectively (F = 1.53; df = 17,36; p=<0.001). The highest mortality (%) was observed with the *Artemisia absinthium* plant extract (87.63 ± 4.21%) which was followed by *Acorus calamus* (83.23 ± 4.02%), *Digitalis purpurea* (64.82 ± 3.59%), *Plectranthus rugosus* (56.04 ± 3.21%) and *Achiella millefolium* (51.88 ± 3.15%) at 72 hrs after treatment (F = 2.02; df = 17,36; p= < 0.001). The mortality observed with the two positive controls was 90.31 ± 4.47 (Deltamethrin 2.5 SC) and 87.64 ± 4.27% (Lambda-cyhalothrin 4.9 CS) at 72 hrs after treatment. Further, low insecticidal activity (2.53 ± 0.27) was observed in the negative control.

**Fig 2:**
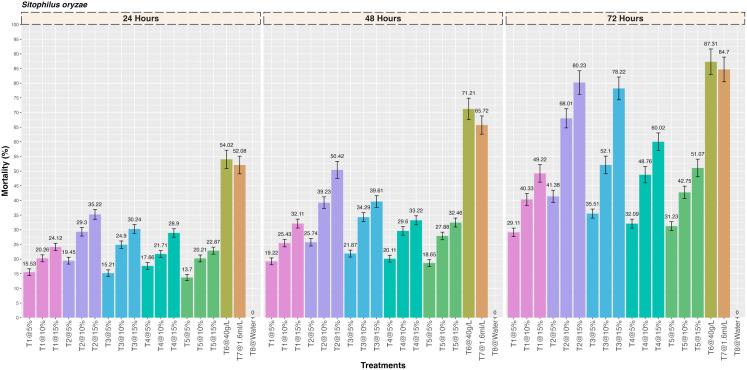
Insecticidal activity of five different medicinal plant extracts against rice weevil, *Sitophilus oryzae* by contact toxicity at different concentrations.

Significant differences were observed between the treatments with respect to the mortality of *S. oryzae* larvae caused by five different medicinal plant extracts at three different concentrations (5%, 10% and 15%) (Fig 2). A significant increase in the mortality with increase in the concentration of each plant extract was observed and *Artemisia absinthium* plant extract, *Acorus calamus, Digitalis purpurea, Plectranthus rugosus* and *Achiella millefolium* caused 35.22 ± 1.67, 30.24 ± 1.50, 28.90 ± 1.44, 22.87 ± 1.21 and 24.12 ± 1.26% mortality at 24 hrs after treatment, respectively (F = 5.65; df = 17,36; p = < 0.001). Similarly, the mortality observed at 48 hrs after treatment was 50.42 ± 2.89, 39.61 ± 1.99, 33.22 ± 1.54, 32.46 ± 1.53 and 32.11 ± 1.52% for *Artemisia absinthium*, *Acorus calamus, Digitalis purpurea, Plectranthus rugosus* and *Achiella millefolium* plant extract, respectively (F = 8.02; df = 17,36; p=<0.001). The highest mortality (%) was observed with the *Artemisia absinthium* plant extract (80.23 ± 4.02) which was followed by *Acorus calamus* (78.22 ± 3.89), *Digitalis purpurea* (60.02 ± 3.01), *Plectranthus rugosus* (51.07 ± 3.01) and *Achiella millefolium* (49.22 ± 2.99) at 72 hrs after treatment (F = 1.60; df = 17,36; p= < 0.001). The mortality observed with the two positive controls was 87.31 ± 4.37% (Deltamethrin 2.5SC) and 84.70 ± 4.21% (Lambda-cyhalothrin 4.9 CS) at 72 hrs after treatment. Further, no insecticidal activity was observed in the negative control.

**Fig 3:**
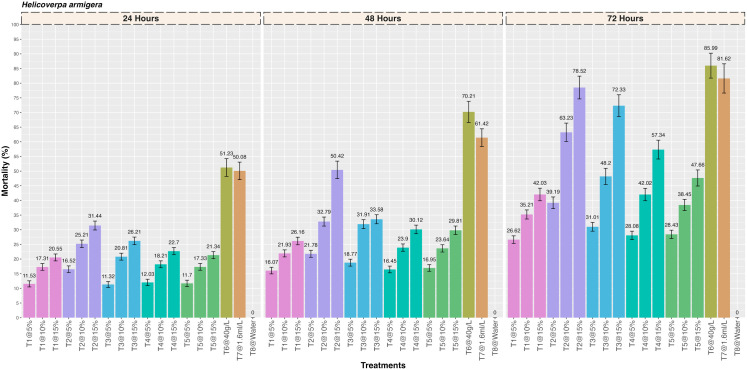
Insecticidal activity of five different medicinal plant extracts against gram pod borer, *Helicoverpa armigera* by contact toxicity at different concentrations.

Observations on the insecticidal activity of five different medicinal plant extracts against *H. armigera* larvae revealed significant differences in the mortality at three different concentrations (5%, 10% and 15%) (Fig 3). It was found that there was a significant increase in the mortality with increase in the concentration of each plant extract and the mortality observed with the *Artemisia absinthium* plant extract, *Acorus calamus, Digitalis purpurea, Plectranthus rugosus* and *Achiella millefolium* was 31.44 ± 1.52, 26.21 ± 1.29, 22.70 ± 1.21, 21.34 ± 1.19 and 20.55 ± 1.18% at 24 hrs after treatment, respectively (F = 5.38; df = 17,36; p=<0.001). Similarly, the mortality observed at 48 hrs after treatment was 50.42 ± 2.99, 33.58 ± 1.54, 30.12 ± 1.46, 29.81 ± 1.45 and 26.16 ± 1.29% for *Artemisia absinthium, Acorus calamus, Digitalis purpurea, Plectranthus rugosus* and *Achiella millefolium* plant extract, respectively (F = 8.81; df = 17,36; p=<0.001). The highest mortality (%) was observed with the *Artemisia absinthium* plant extract (78.52 ± 3.89) which was significantly different as compared to the *Acorus calamus* (72.33 ± 3.72), *Digitalis purpurea* (57.34 ± 3.21), *Plectranthus rugosus* (47.66 ± 2.74) and *Achiella millefolium* (42.03 ± 2.15) at 72 hrs after treatment (F = 1.67; df = 17,36; p= < 0.001). The mortality observed with the two positive controls was 85.99 ± 4.25% (Deltamethrin 2.5 SC) and 81.62 ± 4.99% (Lambda-cyhalothrin 4.9 CS) at 72 hrs after treatment. Further, no mortality was observed in the negative control.

### Binding free energy values of various small molecules derived from *Artemisia absinthium* extract against acetylcholinesterase of *Helicoverpa armigera*

**Fig 4:**
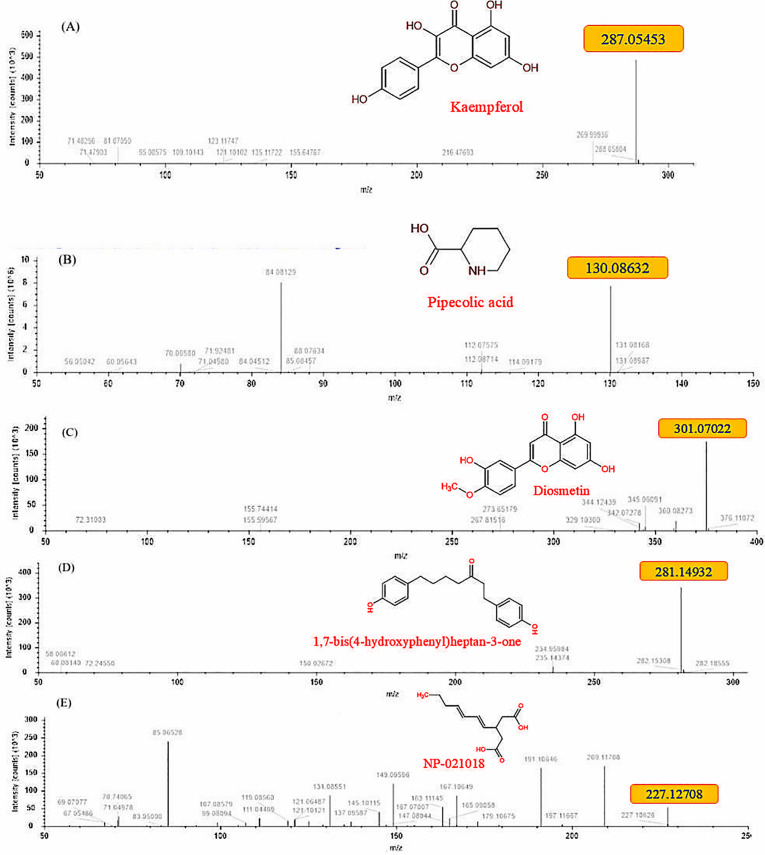
Diverse molecules identified from the extract of *Artemisia absinthium* through HR-. LCMS. Among the explored molecules kaempferol (A), pipecolic acid (B), diosmetin (C) and two others (D, E) were prominent.

**Fig 5:**
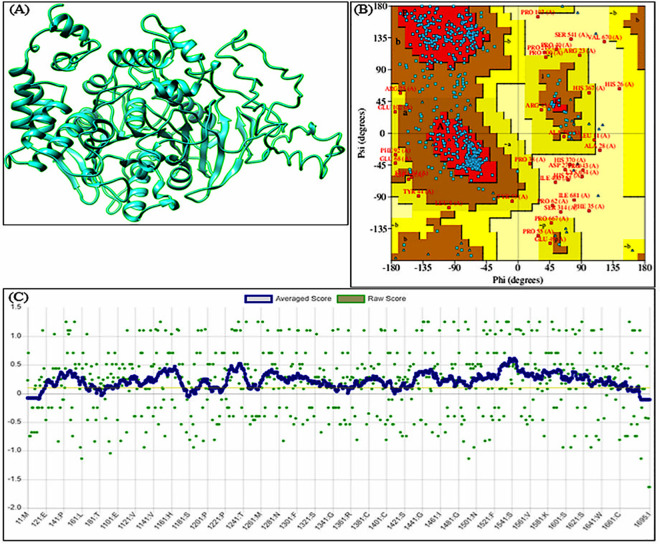
Three-dimensional model of acetylcholinesterase of *Helicoverpa* armigera (A) and its validation through Ramachandran plot (B) and Verify 3D (C).

**Fig 6:**
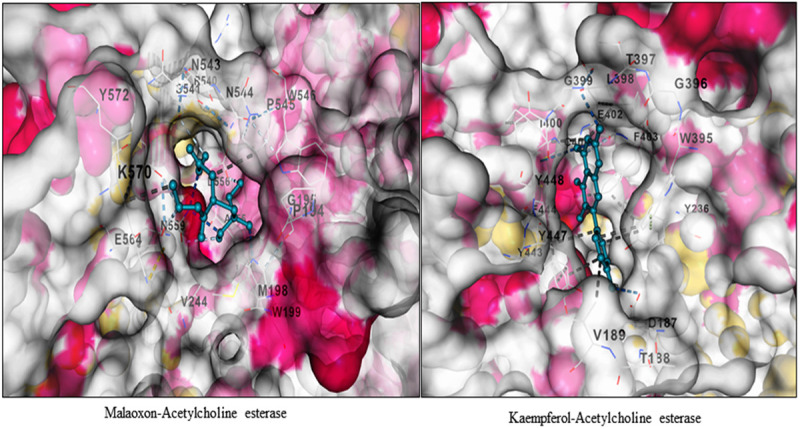
Residues of acetylcholinesterase interacting with malaoxon (A) and kaempferol (B). Both malaoxon and kaempferol bind at the same site of the said esterase.

Using proper solvents and strict procedure a variety of molecules including kaempferol, diosmetin and pipecolic acid were confirmed from the extract (Fig 4A–E). The refined model passed the stereochemical quality check as 77.8% residues were found in most favored regions, 17.6% in additional allowed regions, 2.5% and 2.0% residues in generously allowed regions and disallowed regions, respectively. Further, the model passed the 3D/1D compatibility test successfully as 83.60% of its residues evinced mean 3D-1D score ≥ 0.1 (Fig 5A–C). This is because the minimum limit of the model to pass this test is that 80% of its residues should show 3D-1D score ≥ 0.1 [29,24]. Molecular docking study demonstrated that among the five molecules confirmed from the plant extract, four have more negative binding free energy (more affinity) than the reference molecule (Malaoxon) against the said acetylcholinesterase. While the binding free energy of Malaoxon was estimated to be −5.6 kcal/mol, the defined energy of Kaempferol was quantified to be −9.0 kcal/mol. Diosmetin, 1,7-Bis(4-hydroxyphenyl) heptan-3-one and NP-021018 exhibited the aforementioned value as −8.6, −8.7 and −6.9 kcal/mol, respectively. Only Pipecolic acid manifested a poor binding affinity (−5.2 kcal/mol) than Malaoxon. On the whole among the *Artemisia absinthium* derived molecules Kaempferol demonstrated the highest binding affinity towards acetylcholinesterase, followed by 1,7-Bis(4-hydroxyphenyl) heptan-3-one, Diosmetin and NP-021018 (Table 1). This crux is derived as the lowest binding free energy value infers stronger binding affinity and *vice versa* [30,31]. The interaction profile of positive control and Kaempferol in docked state with acetylcholinesterase was generated which indicated that both the ligands interacted with the multiple residues of this esterase. Malaoxon showed interactions with Gly195, Pro194, Met198, Val244, Trp199, Val244, Arg540, Ser541, Asn543, Asn544, Pro545, Trp546, Ala555, Asp556, Asn559, Glu564, Lys570 and Tyr572. On the other hand Kaempferol evinced interactions with Asp187, Tyr236, Trp395, Gly396, Thr397, Leu398, Ile400, Cys401, Glu402, Phe402, Tyr443, Phe444, Tyr447 and Tyr448 (Fig 6). Thus it is clear that Malaoxon and Kaempferol showed differential interaction profile which may possibly be ascribed to the significant structural diversity between them.

**Table 1:** Binding free energy values of various small molecules derived from *Artemisia absinthium* extract against acetylcholinesterase of *Helicoverpa armigera.*

Ligand	Receptor	Cavity volume (Å^3^)	Center (x, y, z)	Binding free energy (kcal/mol)
Kaempferol	*Helicoverpa armigera* acetylcholine esterase	3301	72, 63, 78	−9.0
Pipecolic acid				−5.2
Diosmetin				−8.6
1,7-Bis(4-hydroxyphenyl)heptan-3-one				−8.7
NP-021018				−6.9
Malaoxon*				−5.6

* signifies positive control

## Discussion

Extensive and indiscriminate use of synthetic pesticides have posed several problems like toxic residues, environmental pollution, health hazardous to the personnel involved in their application, pest resurgence and resistance in insect pests. There are more than 980 species of insect pests reported to have developed resistance against at least 101 active ingredients [32]. In this context, the botanical pesticides could be an alternative to chemical pesticides owing to their safety to non-target organisms, wide availability, inexpensive and rapid biodegradation. The botanical pesticides have been developed and evaluated in India and abroad and they have shown promising results against many insect pests. The use of Neem (*Azadirachtica indica*), Pyrethrum (*Chrysanthemum cinerariifolium*), wild marigold (*Targetes minuta*), yellow sage (*Lantana camara*), sabadilla (*Ryania speciose*), garlic (*Allium sativum*) and other botanicals as biorational pesticides have been encouraged for a reduction in pesticide use in the environment and they are also fit for use in the organic farming. In Jammu and Kashmir the insecticidal activity of many botanicals such as nirgundi (*Vitex negundo*), vasaka (*Justicia adhatoda*), oleander (*Nerium indicum*), wormwood (*Artemisia absinthium*), sweet flag (*Acorus calamus*), common yarrow (*Achiella millefolium*), jimsonweed (*Datura stramonium*) common foxglove (*Digitalis purpurea*) and ginger (*Zingiber officinale*) have been experimentally proved and efforts are being made to commercialize them as biorational pesticides as reported by Hamada *et al*. (2018); Rajput *et al*. (2018); Alkan, (2020) [33–35]. Based on these facts, the insecticidal activities of extracts from five different temperate medicinal plants was evaluated against *Corcyra cephalonica*, *Sitophilus oryzae* and *Helicoverpa armigera* under laboratory conditions and the most potent extract was characterized and its molecular docking against acetylcholinesterase was conducted through computational approaches.

The five different medicinal plant extracts caused mortality in the *C. cephalonica, S. oryzae* and *H. armigera* larvae at three different concentrations and significantly higher mortality was observed with the *A. absinthium* plant extract which was followed by *A. calamus*, *D. purpurea*, *P. rugosus* and *A. millefolium* at 72 hrs after treatment. However, the mortality (%) varied significantly with respect to the host and *A. absinthium* caused 87.63% mortality in *C. cephalonica*, 80.23% in *S. oryzae* and 78.52% mortality in *H. armigera* larvae at 72 hrs after treatment. The descending order of the insecticidal activity of extracts from five different medicinal plants was *A. absinthium*>*A. calamus*>*D. purpurea*>*P. rugosus*>*A. millefolium*. The insecticidal activity of *A. absinthium* is to due the presence of major compound α-thujone and some other compounds such as Camphor, 1,8 cineole and Camphene [36]. Besides, there are several species of *Artemisia* and the concentration of α-thujone in their essential oil and their corresponding insecticidal activity varied significantly [37].

Acetylcholinesterase (AChE) has been a key target for insecticide development as its inhibition stops nerve transmission at synapses. Based on the results of insecticidal activity data, the most potent extract, i.e., *A. absinthium* was characterized and its molecular docking was carried out against acetylcholinesterase enzyme of *Helicoverpa armigera*. The small molecules identified in the *A. absinthium* extract were Kaempferol, Pipecolic acid, Diosmetin 1,7-Bis(4-hydroxyphenyl) heptan-3-one and NP-021018 using HR-LCMS. The molecular docking study revealed that the four molecules namely Kaempferol, Diosmetin, 1,7-Bis(4-hydroxyphenyl) heptan-3-one and NP-021018 have more negative binding free energy (more affinity) than the reference molecule (Malaoxon) against *Helicoverpa armigera* acetylcholinesterase. The pipecolic acid manifested a poor binding affinity (−5.2 kcal/mol) than Malaoxon against *Helicoverpa armigera* acetylcholinesterase. Our results are in line with the previous studies in which the insecticidal activity of methanolic extracts of *Artemisia absinthium* and two cupressaceae species, namely phoenicean juniper (*Juniperus phoenicea*) and arar (*Tetraclinis articulate*) against rice weevil, *S. oryzae* were determined by Dane *et al*. (2016) [38]. The extracts were analyzed by Ultra Performance Liquid Chromatography-Photodiode Array Detection-Mass Spectrometry (UPLC-PDA-MS) and the results showed the presence of several phenolic acids in *A. absinthium* and flavonoids in two cupressaceae species. The study conducted by Liu *et al*. (2021) [28] revealed that the *Artemisia nakaii* essential oil contains 20 compounds, primarily composed of monoterpenes and sesquiterpenes. The most abundant terpenes identified were Feropodin, (+)-camphor, β-selinene and 1,8-cineole and they exhibit potent fumigant toxicity and antifeedant effects against *Spodoptera litura* larvae. Further, they reported that the *Artemisia nakaii* essential oil is effective in inhibiting acetylcholinesterase enzyme of *S. litura*. The homology modelling of the AChE of the aphids and beetles was conducted using SWISS-MODEL and the phytocompounds of congress grass (*Parthenium hysterophorus*), lantana (*Lantana camara*) and sessile joy weed (*Alternanthera sessilis*) were used as ligand molecules [39]. The docking results showed that Kaempferol exhibited least binding energy with the proteins of grain aphid (*Rhopalosiphum padi*), pea aphid (*Acyrthosiphon pisum*) and green peach aphid (*Myzus persicae*). Besides, the phytocompounds of *L. camara* showed least binding energy when docked with AChE of Colorado potato beetle (*Leptinotarsa decemlineata*) and bull-headed dung beetle (*Onthophagus taurus*).

On the basis of molecular docking study, the Malaxon (positive control) and Kaempferol showed different interaction profile with acetylcholinesterase of *Helicoverpa armigera* which indicated that both the ligands interacted with the multiple residues of this esterase. Therefore, it’s concluded that the Kaempferol derived from *Artemisia absinthium* have the highest binding affinity towards *Helicoverpa armigera* acetylcholinesterase. It’s concluded that all the plant extracts described in this study possess significant insecticidal properties and can be developed as botanical insecticides following rigorous field level studies.

## Supporting information

S1 Table Insecticidal activity of five different medicinal plants against rice moth, *Corcyra cephalonica* by contact toxicity at different concentrations. (DOCX)

S2 Table Insecticidal activity of five different medicinal plants against rice weevil, *Sitophilus oryzae* by contact toxicity at different concentrations. (DOCX)

S3 Table Insecticidal activity of five different medicinal plants against gram pod borer, *Helicoverpa armigera* by contact toxicity at different concentrations. (DOCX)
