# Supplementary material for: Insecticidal potential of five medicinal plants: An In Vitro evaluation and molecular docking analysis of Artemisia absinthium
Source: PLoS One. 2025 Jul 1;20(7):e0325959. doi: 10.1371/journal.pone.0325959 (PMC12212477; doi:10.1371/journal.pone.0325959)
Supplement: S1 Table — (DOCX) [file pone.0325959.s001.docx]

**S1 Table:** Insecticidal activity of five different medicinal plants against rice moth, *Corcyra cephalonica* by contact toxicity at different concentrations

| Treatments | Concentration | Mortality (%)  (Mean ± SE) | | |
| --- | --- | --- | --- | --- |
|  |  | 24hrs | 48hrs | 72hrs |
| T1 (*Achiella millefolium*) | 5% | 16.94±1.14^b^ | 20.42±1.23^b^ | 33.51±1.85^b^ |
| T2 ( *Achiella millefolium*) | 10% | 22.09±1.25^d^ | 29.53±1.43^f^ | 46.22±2.11^g^ |
| T3 ( *Achiella millefolium*) | 15% | 29.12±1.41^h^ | 35.10±1.89^i^ | 51.88±3.15^i^ |
| T4 (*Artemesia absinthium*) | 5% | 22.23±1.27^d^ | 29.34±1.45^f^ | 45.39±2.07^f^ |
| T5 (*Artemesia absinthium*) | 10% | 31.36±1.35^i^ | 42.46±2.11^l^ | 72.44± 3.65^m^ |
| T6 (*Artemesia absinthium*) | 15% | 39.82±1.79^k^ | 53.33±3.19^n^ | 87.63±4.21^o^ |
| T7 (*Acorus calamus*) | 5% | 18.33±1.18^c^ | 25.66±1.29^e^ | 40.33±2.01^e^ |
| T8 (*Acorus calamus*) | 10% | 27.66±1.31^g^ | 37.43±1.92^j^ | 57.20±3.21^k^ |
| T9 (*Acorus calamus*) | 15% | 34.54±1.76^j^ | 43.24±2.06^m^ | 83.23±4.02^n^ |
| T10 (*Digitalis purpurea*) | 5% | 17.66±1.16^c^ | 23.13±1.22^d^ | 37.17±1.92^d^ |
| T11 (*Digitalis purpurea*) | 10% | 24.45±1.32^f^ | 33.21±1.36^h^ | 52.23±3.19^j^ |
| T12 (*Digitalis purpurea*) | 15% | 31.46±1.36^i^ | 39.20±0.04^k^ | 64.82±3.59^l^ |
| T13 (*Plectranthus rugosus*) | 5% | 16.66±1.11^b^ | 21.63±1.23^c^ | 35.29±1.91^c^ |
| T14 (*Plectranthus rugosus*) | 10% | 23.21±1.29^e^ | 30.11±1.31^g^ | 49.86±2.54^h^ |
| T15 (*Plectranthus rugosus*) | 15% | 29.87±1.45^h^ | 37.26±1.91^j^ | 56.04±3.21^k^ |
| T16 (Deltamethrin 2.5 SC) | 0.0025%  (1 ml/L) | 56.32±3.26^l^ | 75.21±3.77^p^ | 90.31±4.47^p^ |
| T17 (Lambda-cyhalothrin  4.9 CS) | 0.0078%  (1.6 ml/L) | 58.08±3.25^m^ | 69.72±3.29^o^ | 87.64±4.27^o^ |
| T18 (Distilled water) | - | 0.00±0.00^a^ | 0.00±0.00^a^ | 2.53±0.27^a^ |
| F/df/p | | 7.27/17,36/<0.001 | 1.53/17,36/<0.001 | 2.02/17,36/<0.001 |

Mean ± SE followed by different superscripts within the same column are significantly different at p < 0.05.
