# Supplementary material for: Insecticidal potential of five medicinal plants: An In Vitro evaluation and molecular docking analysis of Artemisia absinthium
Source: PLoS One. 2025 Jul 1;20(7):e0325959. doi: 10.1371/journal.pone.0325959 (PMC12212477; doi:10.1371/journal.pone.0325959)
Supplement: S2 Table — (DOCX) [file pone.0325959.s002.docx]

**S2 Table:** Insecticidal activity of five different medicinal plants against rice weevil, *Sitophilus oryzae* by contact toxicity at different concentrations

| Treatments | Concentration | Mortality (%)  (Mean ± SE) | | |
| --- | --- | --- | --- | --- |
|  |  | 24hrs | 48hrs | 72hrs |
| T1 (*Achiella millefolium*) | 5% | 15.53±1.13^c^ | 19.22±1.16^c^ | 29.11±1.45^b^ |
| T2 ( *Achiella millefolium*) | 10% | 20.26±1.18^ef^ | 25.43±1.27^e^ | 40.33±2.01^e^ |
| T3 ( *Achiella millefolium*) | 15% | 24.12±1.26^i^ | 32.11±1.52^h^ | 49.22±2.99^g^ |
| T4 (*Artemesia absinthium*) | 5% | 19.45±1.16^e^ | 25.74±1.28^e^ | 41.38±2.12^f^ |
| T5 (*Artemesia absinthium*) | 10% | 29.30±1.45^k^ | 39.23±1.99^j^ | 68.01±3.27^j^ |
| T6 (*Artemesia absinthium*) | 15% | 35.22±1.67^l^ | 50.42±2.89^k^ | 80.23±4.02^l^ |
| T7 (*Acorus calamus*) | 5% | 15.21±1.12^c^ | 21.87±1.19^d^ | 35.51±1.55^d^ |
| T8 (*Acorus calamus*) | 10% | 24.90±1.27^i^ | 34.29±1.54^i^ | 52.10±3.02^h^ |
| T9 (*Acorus calamus*) | 15% | 30.24±1.50^k^ | 39.61±1.99^j^ | 78.22±3.89^k^ |
| T10 (*Digitalis purpurea*) | 5% | 17.66±1.15^d^ | 20.11±1.18^d^ | 32.09±1.52^c^ |
| T11 (*Digitalis purpurea*) | 10% | 21.71±1.19^g^ | 29.60±1.45^g^ | 48.76±2.78^g^ |
| T12 (*Digitalis purpurea*) | 15% | 28.90±1.44^j^ | 33.22±1.54^i^ | 60.02±3.01^i^ |
| T13 (*Plectranthus rugosus*) | 5% | 13.70±1.01^b^ | 18.65±1.15^b^ | 31.23±1.52^c^ |
| T14 (*Plectranthus rugosus*) | 10% | 20.21±1.18^f^ | 27.88±1.30^f^ | 42.75±2.13^f^ |
| T15 (*Plectranthus rugosus*) | 15% | 22.87±1.21^h^ | 32.46±1.53^h^ | 51.07±3.01^h^ |
| T16 (Deltamethrin 2.5 SC) | (0.0025%)  1 ml/L | 54.02±3.12^n^ | 71.21±3.66^m^ | 87.31±4.37^n^ |
| T17 (Lambda-cyhalothrin 4.9 CS) | 0.0078%  (1.6 ml/L) | 52.08±3.02^m^ | 65.72±3.11^l^ | 84.70±4.21^m^ |
| T18 (Distilled water) | - | 0.00±0.00^a^ | 0.00±0.00^a^ | 0.00±0.00^a^ |
| F/df/p | | 5.65/17,36/<0.001 | 8.02/17,36/<0.001 | 1.60/17,36/<0.001 |

Mean ± SE followed by different superscripts within the same column are significantly different at p < 0.05.
