# Supplementary material for: Insecticidal potential of five medicinal plants: An In Vitro evaluation and molecular docking analysis of Artemisia absinthium
Source: PLoS One. 2025 Jul 1;20(7):e0325959. doi: 10.1371/journal.pone.0325959 (PMC12212477; doi:10.1371/journal.pone.0325959)
Supplement: S3 Table — (DOCX) [file pone.0325959.s003.docx]

**S3 Table:** Insecticidal activity of five different medicinal plants against gram pod borer, *Helicoverpa armigera* by contact toxicity at different concentrations

Mean ± SE followed by different superscripts within the same column are significantly different at p < 0.05

| Treatments | Concentration | Mortality (%)  (Mean ± SE) | | |
| --- | --- | --- | --- | --- |
|  |  | 24hrs | 48hrs | 72hrs |
| T1 (*Achiella millefolium*) | 5% | 11.53±1.04^bc^ | 16.07±1.13^b^ | 26.62±1.30^b^ |
| T2 ( *Achiella millefolium*) | 10% | 17.31±1.16^e^ | 21.93±1.20^d^ | 35.21±1.55^e^ |
| T3 ( *Achiella millefolium*) | 15% | 20.55±1.18^f^ | 26.16±1.29^f^ | 42.03±2.15^g^ |
| T4 (*Artemesia absinthium*) | 5% | 16.52±1.14^d^ | 21.78±1.20^d^ | 39.19±1.99^f^ |
| T5 (*Artemesia absinthium*) | 10% | 25.21±1.28^i^ | 32.79±1.54^i^ | 63.23±3.15^k^ |
| T6 (*Artemesia absinthium*) | 15% | 31.44±1.52^k^ | 50.42±2.99^k^ | 78.52±3.89^m^ |
| T7 (*Acorus calamus*) | 5% | 11.32±1.01^bc^ | 18.77±1.18^c^ | 31.01±1.50^d^ |
| T8 (*Acorus calamus*) | 10% | 20.81±1.18^f^ | 31.91±1.52^h^ | 48.20±2.77^i^ |
| T9 (*Acorus calamus*) | 15% | 26.21±1.29^j^ | 33.58±1.54^j^ | 72.33±3.72^l^ |
| T10 (*Digitalis purpurea*) | 5% | 12.03±1.07^c^ | 16.45±1.14^b^ | 28.08±1.43^c^ |
| T11 (*Digitalis purpurea*) | 10% | 18.21±1.17^e^ | 23.90±1.25^e^ | 42.02±2.13^g^ |
| T12 (*Digitalis purpurea*) | 15% | 22.70±1.21^h^ | 30.12±1.46^gh^ | 57.34±3.21^j^ |
| T13 (*Plectranthus rugosus*) | 5% | 11.70±1.08^b^ | 16.95±1.14^b^ | 28.43±1.43^c^ |
| T14 (*Plectranthus rugosus*) | 10% | 17.33±1.16^e^ | 23.64±1.25^e^ | 38.45±1.91^f^ |
| T15 (*Plectranthus rugosus*) | 15% | 21.34±1.19^g^ | 29.81±1.45^g^ | 47.66±2.74^h^ |
| T16 (Deltamethrin 2.5 SC) | (0.0025%)  1 ml/L | 51.23±3.01^m^ | 70.21±3.61^m^ | 85.99±4.25^o^ |
| T17 (Lambda-cyhalothrin 4.9 CS) | 0.0078%  (1.6 ml/L) | 50.08±2.99^l^ | 61.42±3.01^l^ | 81.62±4.99^n^ |
| T18 (Distilled water) | - | 0.00±0.00^a^ | 0.00±0.00^a^ | 0.00±0.00^a^ |
| F/df/p | | 5.38/17,36/<0.001 | 8.81/17,36/<0.001 | 1.67/17,36/<0.001 |
